# SARS-CoV-2-Specific T Cell Responses Are Stronger in Children With Multisystem Inflammatory Syndrome Compared to Children With Uncomplicated SARS-CoV-2 Infection

**DOI:** 10.3389/fimmu.2021.793197

**Published:** 2022-01-18

**Authors:** Susan R. Conway, Christopher A. Lazarski, Naomi E. Field, Mariah Jensen-Wachspress, Haili Lang, Vaishnavi Kankate, Jessica Durkee-Shock, Hannah Kinoshita, William Suslovic, Kathleen Webber, Karen Smith, Jeffrey I. Cohen, Peter D. Burbelo, Anqing Zhang, Stephen J. Teach, Trisha Ibeh, Meghan Delaney, Roberta L. DeBiasi, Michael D. Keller, Catherine M. Bollard

**Affiliations:** ^1^ Center for Cancer and Immunology Research, Children’s National Hospital, Washington, DC, United States; ^2^ Division of Critical Care Medicine, Children’s National Hospital, Washington, DC, United States; ^3^ Department of Pediatrics, George Washington University School of Medicine and Health Sciences, Washington, DC, United States; ^4^ Laboratory of Infectious Diseases, National Institutes of Health, Bethesda, MD, United States; ^5^ Division of Hematology and Oncology, Children’s National Hospital, Washington, DC, United States; ^6^ Division of Pathology and Laboratory Medicine, Children’s National Hospital, Washington, DC, United States; ^7^ Department of Pediatrics, Children’s National Hospital, Washington, DC, United States; ^8^ National Institute of Dental and Craniofacial Research, National Institutes of Health, Bethesda, MD, United States; ^9^ Division of Biostatistics and Study Methodology, Children’s National Hospital, Washington, DC, United States; ^10^ Center for Translational Research, Children’s National Hospital, Washington, DC, United States; ^11^ Department of Microbiology, Immunology, and Tropical Medicine, The George Washington University School of Medicine and Health Sciences, Washington, DC, United States; ^12^ Division of Infectious Diseases, Children’s National Hospital, Washington, DC, United States; ^13^ Division of Allergy and Immunology, Children’s National Hospital, Washington, DC, United States; ^14^ GW Cancer Center, George Washington University, Washington, DC, United States

**Keywords:** SARS-CoV-2, T cell, MIS-C, COVID-19, pediatric

## Abstract

**Background:**

Despite similar rates of infection, adults and children have markedly different morbidity and mortality related to severe acute respiratory syndrome coronavirus-2 (SARS-CoV-2). Compared to adults, children have infrequent severe manifestations of acute infection but are uniquely at risk for the rare and often severe Multisystem Inflammatory Syndrome in Children (MIS-C) following infection. We hypothesized that these differences in presentation are related to differences in the magnitude and/or antigen specificity of SARS-CoV-2-specific T cell (CST) responses between adults and children. We therefore set out to measure the CST response in convalescent adults versus children with and without MIS-C following SARS-CoV-2 infection.

**Methods:**

CSTs were expanded from blood collected from convalescent children and adults post SARS-CoV-2 infection and evaluated by intracellular flow cytometry, surface markers, and cytokine production following stimulation with SARS-CoV-2-specific peptides. Presence of serum/plasma antibody to spike and nucleocapsid was measured using the luciferase immunoprecipitation systems (LIPS) assay.

**Findings:**

Twenty-six of 27 MIS-C patients, 7 of 8 non-MIS-C convalescent children, and 13 of 14 adults were seropositive for spike and nucleocapsid antibody. CST responses in MIS-C patients were significantly higher than children with uncomplicated SARS-CoV-2 infection, but weaker than CST responses in convalescent adults.

**Interpretation:**

Age-related differences in the magnitude of CST responses suggest differing post-infectious immunity to SARS-CoV-2 in children compared to adults post uncomplicated infection. Children with MIS-C have CST responses that are stronger than children with uncomplicated SARS-CoV-2 infection and weaker than convalescent adults, despite near uniform seropositivity.

## Introduction

Severe acute respiratory syndrome coronavirus-2 (SARS-CoV-2) has infected more than 250 million people and caused more than 5 million deaths worldwide. Despite similar infection rates across age groups, the mortality burden has fallen largely upon adults with relatively few acute cases requiring hospitalization among children (1, 2). Sequelae of SARS-CoV-2 infection, including Multisystem Inflammatory Syndrome in Children (MIS-C), also differ between adults and children. The reason for these age-related differences in disease course is not known but likely involves differences in the host response to infection. Therefore, an improved understanding of the pediatric immune response to SARS-CoV-2 has important implications for the treatment and prevention of illness in adults and children.

MIS-C is a life-threatening hyperinflammatory syndrome that occurs in children several weeks after primary SARS-CoV-2 infection and is characterized by the presence of fever, multiorgan involvement—commonly dermatologic, gastrointestinal, and cardiovascular—and markedly elevated inflammatory markers (3–6). While MIS-C bears similarities to Kawasaki Disease (KD), a vasculitis that presents in children, its immunologic profile is distinct, indicating likely differing pathobiology (7–10). Recent studies have shown an association between development of MIS-C and expansion of Vbeta 21.3+ CD4+ and CD8+ T cells, activation of vascular patrolling CX3CR1+ CD8+ T cells, putative autoantibody expression, and decreased numbers of tolerogenic dendritic cells, though its cause remains undefined (7, 11–14). Following the identification of MIS-C, multisystem inflammatory syndrome in adults (MIS-A) was also described as a likely post-infectious inflammatory sequela in adults, though it appears to be rarer than MIS-C and its overlap with severe COVID-19 and pediatric MIS-C is not yet well-understood (15, 16).

T cell responses are essential for viral clearance and prolonged immunity post infection, and multiple studies have demonstrated that SARS-CoV-2 induces a strong and lasting T cell response in adults with coronavirus disease 2019 (COVID-19) that is associated with recovery (17–22). We also previously showed that SARS-CoV-2-specific T cells (coronavirus-specific T cells, CSTs) can be expanded from convalescent adults following mild and moderate COVID-19 (23). These findings demonstrate durable CST responses in convalescent adults. Deep immunophenotyping in adults has shown derangements in many immune cell populations in patients with severe COVID-19, which differ from those seen in MIS-C (7, 9, 11, 12, 24, 25). However, the role of CSTs with regard to virus clearance and pathology in children is not yet clear. Hence, we chose to study the CST response in convalescent adults compared to children with and without MIS-C post SARS-CoV-2 infection. Here we are the first to describe functional differences in CSTs from adults and children with and without MIS-C, suggesting that virus-specific immune responses differ between these groups, which is highly likely to have ramifications for future susceptibility and need for vaccination.

## Methods

### Participants

Peripheral blood mononuclear cells (PBMCs) were obtained from participants recruited from Children’s National Hospital (CNH) (Washington, DC) and the National Institutes of Health under informed consent approved by the Institutional Review Board of both institutions in accordance with the Declaration of Helsinki. Participants were eligible for recruitment if they had a history of SARS-CoV-2 infection (based on positive antibody or SARS-CoV-2 PCR) or had a current confirmed or probable diagnosis of MIS-C (6, 26). Probable and confirmed MIS-C were defined according to CNH criteria whereby confirmed MIS-C met the CDC case definition including detectable SARS-CoV-2 antibody, positive SARS-CoV-2 RT-PCR, and/or identified laboratory-confirmed contact within the past 4 weeks (26), and probable MIS-C met all CDC criteria except for positive SARS-CoV-2 RT-PCR, antibody test, or known contact, but with appropriate temporal onset following surge in the community. Clinical data were collected by interview with participants/guardians and retrospectively by medical record review for patients with MIS-C.

### Generation of SARS-CoV-2–Specific T Cells

CSTs were expanded from PBMCs by a 10-day expansion protocol that has been previously described (27). Briefly, PBMCs were plated in 96-well round bottom plates at 200,000 cells per well and pulsed with a mix of overlapping peptide pools encompassing viral structural proteins (0.5 µg/antigen per 1 x 10^6^ cells) then incubated in media supplemented with IL-4 (400 IU/mL) and IL-7 (10 ng/mL). Cytokines were replenished at day 7 and cells were harvested at day 10 for further testing.

### Flow Cytometry

Lymphocytes were stained with fluorophore-conjugated antibodies against CD3, CD4, CD8, CD56, CD107a, HLA-DR, IFN-γ, TNF-α, TCRab, TCRgd, CD45RO, CCR7, CXCR3, CCR4, CCR6, CD25, and CD127 (Miltenyi Biotec; BioLegend). All samples were acquired on a CytoFLEX cytometer (Beckman Coulter, Brea, CA). Intracellular cytokine staining was performed as follows: cells were stimulated with individual peptides (200 ng per peptide per well) or actin (control) in the presence of brefeldin A (Golgiplug; BD Biosciences, San Jose, CA) and CD28/CD49d (BD Biosciences) for 4 hours. T-cells were fixed, permeabilized with Cytofix/Cytoperm solution (BD Biosciences) and stained with IFN-γ and TNF-α antibodies (Miltenyi Biotec). SARS-CoV-2-specific T cells were identified by TNF-α and IFN-γ positivity in response to SARS-CoV-2 peptides.

### Multiplex Cytokine Assay

CSTs post 10-day expansion were plated at 1 x 10^5^ cells per well in 96-well plates then stimulated with pooled pep-mixes (200 ng/peptide/well) or control actin peptide and incubated for 48 hours. Supernatants were harvested and the cytokine profile analysis was performed using the Bio-Plex Pro Human 17-Plex Cytokine Assay Kit (Bio-Rad, Hercules, CA), and read on a MAGPIX system (Luminex, Austin, TX).

### Serology

Quantitative measurements of SARS-CoV-2 spike and nucleocapsid antibodies from participant plasma or serum were made using the luciferase immunoprecipitation assay systems (LIPS) as described previously (28).

### Human Leukocyte Antigen (HLA) Sequencing

Samples were sent for high resolution SSO HLA typing (The Sequencing Center, Fort Collins, CO).

### Statistical Analysis

Statistical analysis was performed in GraphPad Prism 9 (Graphpad Software, San Diego, CA, USA). Data were described using geometric means and medians with 95% confidence intervals. The Mann-Whitney U test was utilized for pairwise analysis of antibody and T cell responses.

## Results

### Study Population

Subjects included 35 previously healthy children—27 hospitalized for MIS-C and 8 with recent uncomplicated SARS-CoV-2 infection (median 84 days since onset of symptoms or positive SARS-CoV-2 PCR)—and 14 adults with a recent mild to moderate SARS-CoV-2 infection (median 164 days since onset of symptoms or positive SARS-CoV-2 PCR) (23). None of the pediatric subjects without MIS-C were hospitalized for their SARS-CoV-2 infections. One was asymptomatic and seven experienced mild symptoms including fever, cough, congestion, headache, and/or diarrhea for up to 1 week. Of the 14 adult subjects, one was hospitalized for severe COVID-19. The other 13 adults experienced mild to moderate symptoms including congestion, cough, fatigue, fever and anosmia lasting 2 days to 2 weeks.

In the MIS-C cohort, 26% (n=7) of patients identified as black or African American and 63% (n=17) identified as of Hispanic, Latino or Spanish origin, while 37.5% (n=3) in the non-MIS-C cohort identified as black or African American and 13% (n=1) identified as Hispanic/Latino/Spanish origin (**Table 1**). Traditional human leukocyte antigen (HLA) types identified in children with (n=21) and without (n=7) MIS-C were consistent with the ethnicity of the population (**Supplemental Table 1**).

**Table 1:** Subject Demographics.

	Age (years)	Gender		Race/Ethnicity			
		F	M	African American or Black	Hispanic/Latino/Spanish Origin	Caucasian	Other
MIS-C	8.7 ± 5.2	48.1%	51.9%	26% (n = 7)	63% (n = 17)	7% (n = 2)	4% (n = 1)
Pediatric non-MISC	5.2 ± 4.3	62.5%	37.5%	37.5% (n = 3)	13% (n = 1)	50% (n = 4)	none
Adult	38.3 ± 12	71.4%	28.6%	none	none	71.4% (n = 10)	28.6%(n = 4)

**Figure 1:**
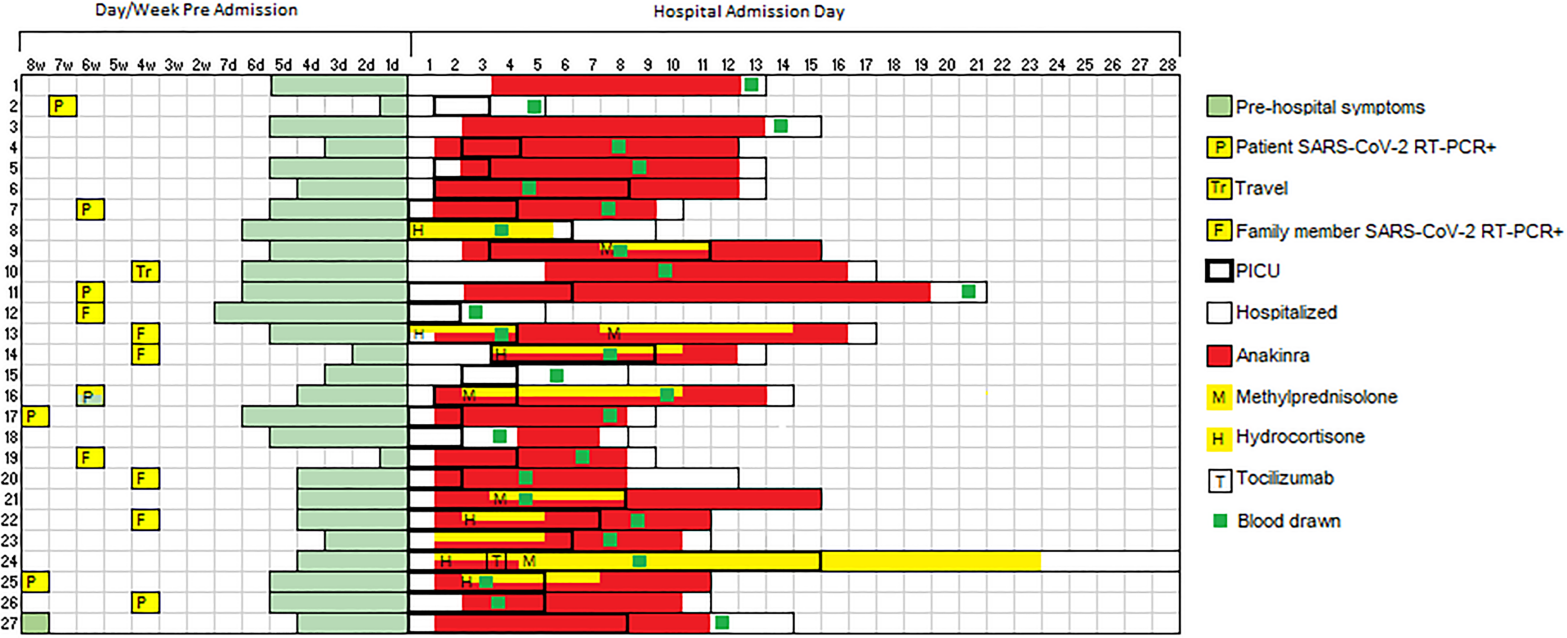
SARS-CoV-2 exposure and MIS-C treatment relative to sample collection. Swimmer plot charting timing of SARS-CoV-2 exposure, symptoms of MIS-C prior to hospitalization, hospital admission, ICU admission, and MIS-C treatment relative to study blood draw. P, patient SARS-CoV-2 RT-PCR positive; F, family member(s) SARS-CoV-2 RT-PCR positive; Tr, travel; H, hydrocortisone; M, methylprednisolone; T, tocilizumab.

Among patients with MIS-C, 89% (n = 24) required intensive care for shock. This represents a high-acuity group of MIS-C patients, of whom approximately 50% generally require critical care at our institution (6). Sixty-three percent (n = 17) required vasopressors for hypotension and 48% were treated for respiratory failure with either non-invasive positive pressure ventilation (n = 8) or invasive mechanical ventilation (n = 5). Consistent with prior reports, a predominance (52%, n = 14) of MIS-C patients showed cardiac involvement on echocardiogram including depressed left ventricular (LV) systolic function (n = 9), mild LV or RV dilation (n = 5), mild valvular insufficiency (n=4), and/or coronary artery ectasia (n = 4). The median peak troponin and BNP were markedly elevated at 0.16 (IQR 0.095-0.75) and 13828 (IQR 6862-22561) pg/mL, respectively (**Table 2**). There was evidence of marked hyperinflammation with a median peak CRP of 16.6 (IQR 12.3-22.9) mg/dL and serum soluble IL-2 receptor, IL-6, and IL-10 of 9002.6 (IQR 6006-11617), 51.4 (IQR 20.1-122), and 182 (IQR 74.0-377) pg/mL, respectively. All patients with MIS-C received at least one dose of intravenous immunoglobulin (IVIG) and 23 patients received the IL-1 receptor antagonist anakinra (6-10 mg/kg/day). Seven patients received intravenous hydrocortisone (50 mg/m^2^/day) for shock and five received methylprednisolone (2-3 mg/kg/day). Among patients treated with methylprednisolone, two samples were drawn after steroid initiation (6-7 days post initiation). Among patients treated with anakinra, 22 of 23 samples were drawn after anakinra initiation (median 6 days post anakinra initiation). Timing of PBMC collection with respect to MIS-C treatment and ICU admission are shown in **Figure 1**. Ten patients with MIS-C reported a possible exposure, most commonly a symptomatic or confirmed infection in a family member, and 7 of 27 reported a positive COVID RNA test 1-2 months prior to presentation with MIS-C (**Figure 1**), timing consistent with prior reports (3, 4, 8). All were asymptomatic or had mild symptoms at that time. Four patients (14.8%) had positive nasal SARS-CoV-2 PCR tests on presentation with MIS-C.

**Table 2:** MIS-C subject clinical characteristics.

	N (% of MIS-C subjects)
ICU admission	24 (89%)
Vasopressor requirement	17 (63%)
Non-invasive positive pressure	8 (30%)
Invasive mechanical ventilation	5 (18.5%)
Abnormal echocardiogram	14 (52%)
Depressed LV systolic function	9 (33%)
LV/RV dilation	5 (18.5%)
Valvular insufficiency	4 (14.8%)
Coronary artery ectasia	4 (14.8%)
	**Median (IQR)**
Duration of invasive mechanical ventilation	2 (IQR 2-4) days
ICU length of stay	4.5 (IQR 2-6.3) days
Peak troponin	0.16 (IQR 0.095-0.75) pg/mL
Peak BNP	13828 (IQR 6862-22561) pg/mL

### The Majority of Pediatric and Adult Subjects Showed SARS-CoV-2 Seropositivity

**Figure 2:**
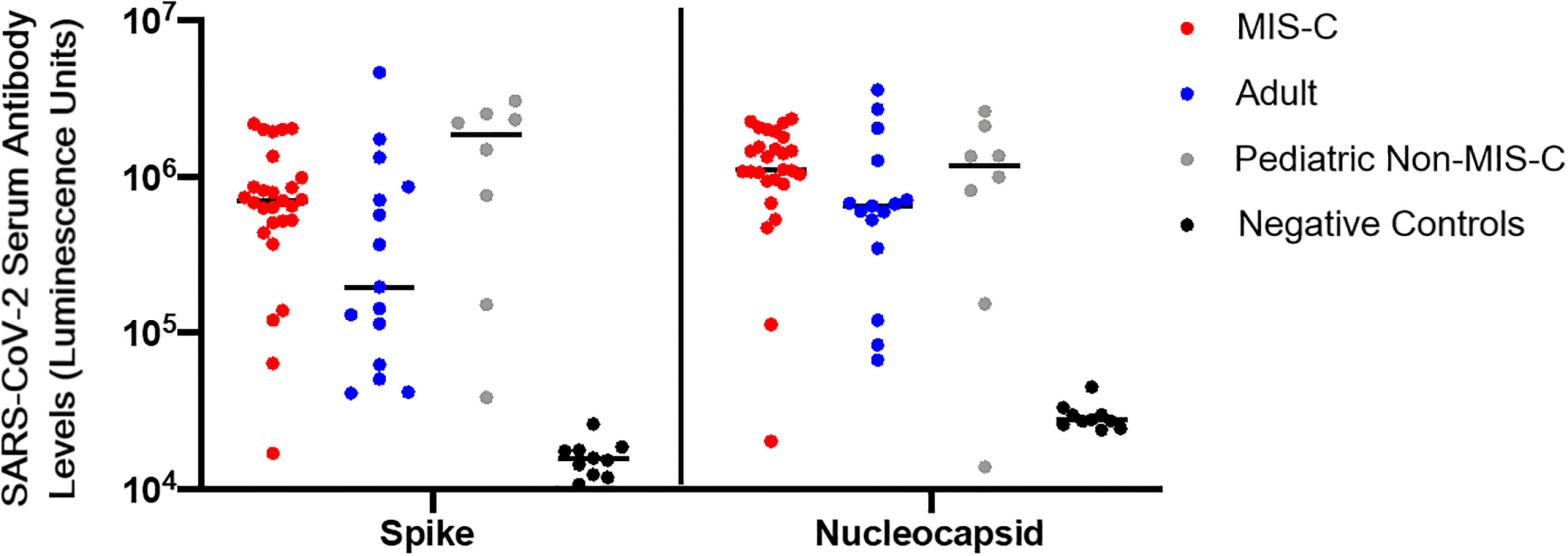
SARS-CoV-2 serology. Serologies in luminescence units for antibodies to SARS-CoV-2 spike and nucleocapsid proteins from adult convalescent (blue), pediatric convalescent (gray), and MIS-C (red) subjects, and negative controls (black). Negative controls are pre-pandemic adult samples. Assays were conducted in duplicate on 10uL of 1:10 diluted plasma. Black line represents the median value for each cohort.

Nearly all patients with MIS-C (n = 26) had evidence of seropositivity to SARS-CoV-2 spike and nucleocapsid proteins by LIPS assay. Seven of 8 (87.5%) pediatric convalescent subjects without MIS-C showed evidence of seropositivity (median 84 [IQR 55-156] days since symptom onset or positive SARS-CoV-2 PCR), and 13 of the 14 (92.9%) adult subjects were seropositive (median of 164 [IQR 102-172] days since symptom onset or positive SARS-CoV-2 PCR) (**Figure 2**). There were no significant differences in the quantity of antibody detected across groups.

### Magnitude of T Cell Responses to SARS-CoV-2 Is Higher in Adult Convalescent Subjects and Children With MIS-C Compared to Children Post Uncomplicated SARS-CoV-2 Infection

**Figure 3:**
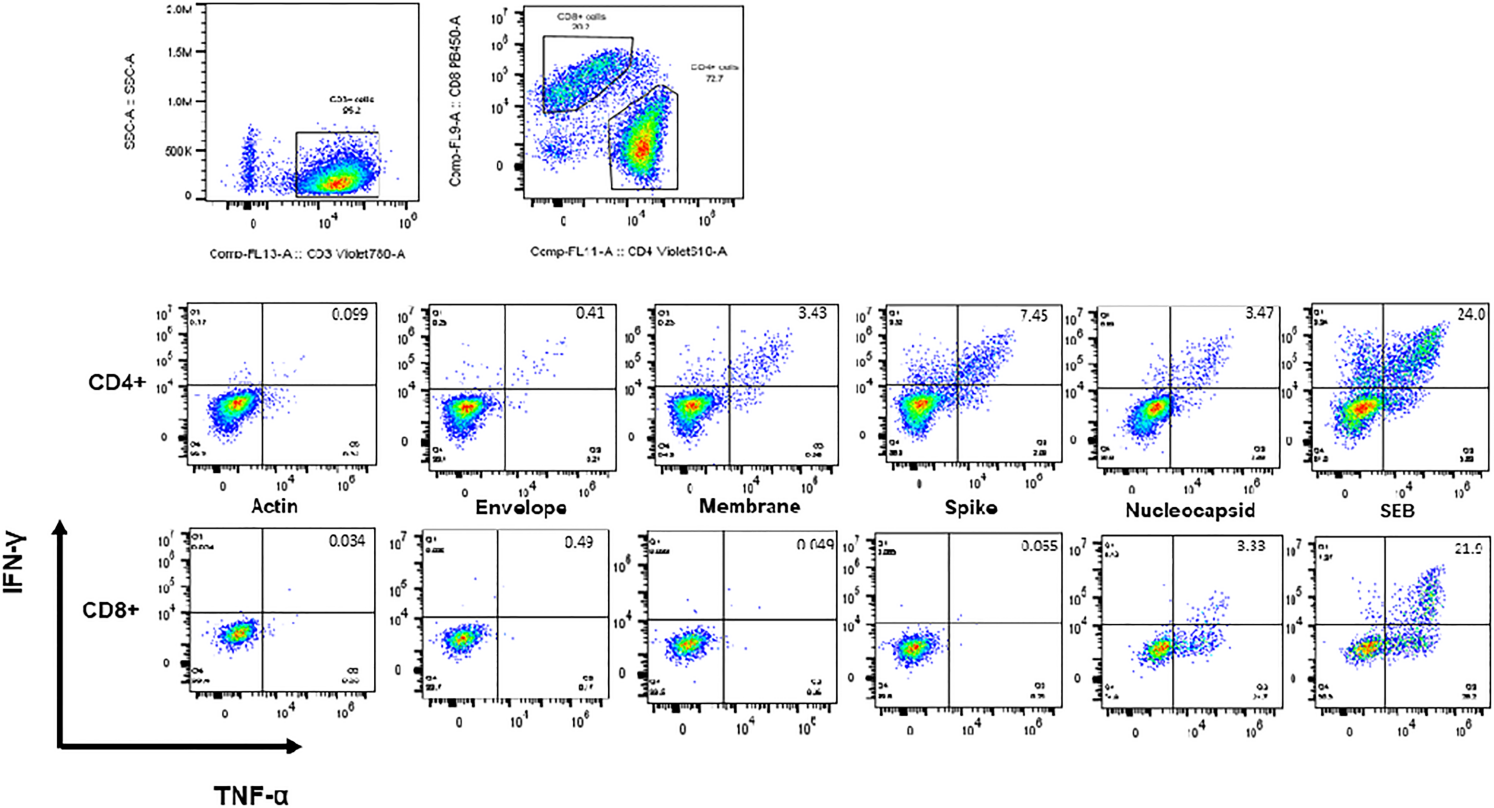
Intracellular staining and flow cytometry. Flow cytometric dot plots from a representative CST population expanded from adult convalescent PBMCs showing CD4+ and CD8+ T cells secreting IFN-γ and TNF-α in response to viral antigen.

**Figure 4:**
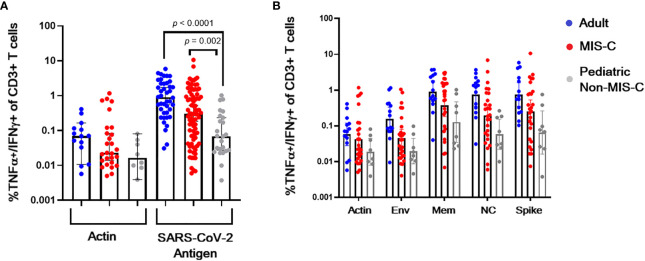
CST response in adults and children with and without MIS-C. **(A)** Percent of CD3+ T lymphocytes from convalescent adult (blue), non-MIS-C pediatric (gray), and MIS-C (red) subjects secreting TNF-α and IFN-γ when stimulated with actin (negative control) or viral antigen post *ex vivo* expansion in the presence of viral peptides. **(B)** Membrane (mem), nucleocapsid (NC), and spike peptides induce TNF-α and IFN-γ CST secretion. Columns, medians, error bars, 95% confidence intervals.

CSTs (based on TNF-α and IFN-γ secretion in response to spike, membrane, or nucleocapsid viral peptides) were readily expanded from convalescent adult (median 0.898% of CD3+ T lymphocytes v 0.07% in actin control, *p* < 0.0001) and MIS-C subjects (median 0.30% of CD3+ T lymphocytes v 0.02% in actin control, *p* < 0.0001) (**Figure 3**). Pediatric subjects without MIS-C yielded a less robust CST population (median 0.069% of CD3+ T lymphocytes versus 0.0.016% in actin control, *p* = 0.02) (**Figure 4A**). This difference in CST response between convalescent adult and pediatric subjects was significant (median 0.898% of CD3+ T lymphocytes in adults versus 0.069% in children without MIS-C, *p* < 0.0001 and 0.302% in children with MIS-C, *p* = 0.0006). Moreover, the CST response in children with MIS-C was significantly higher than that in children without MIS-C (median 0.302% in MIS-C versus 0.069% in non-MIS-C samples, *p* = 0.002). When we analyzed CSTs as a percentage of the CD4^+^ T cell subset these differences were maintained (**Supplemental Figure 1A**). Among CD8^+^ T cells, there was no significant increase in TNF-α and IFN-γ secretion in response to viral antigen compared to the actin negative control, and there were no significant differences between groups (**Supplemental Figure 1B**). CSTs responded to spike, membrane, and nucleocapsid peptides (**Figure 4B**) consistent with previously reported adult data identifying the immunogenicity of epitopes in these proteins (19, 23, 29). Treatment with IL-1 blockade did not affect CST expansion from MIS-C samples (**Supplemental Figure 2**).

### SARS-CoV-2 T Cells Were Polyfunctional and Predominantly CD4+ With Higher Proportions of Central Memory T Cells Detected in MIS-C Patients Compared to Convalescent Adults

**Figure 5:**
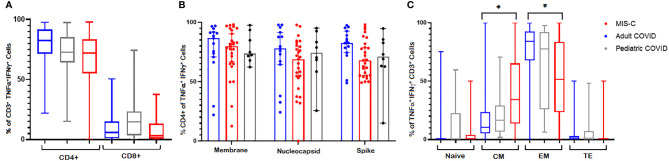
CST Phenotypes. CSTs were predominantly CD4+ across **(A)** cohorts and **(B)** SARS-CoV-2 antigens. **(C)** CSTs expanded from patients with MIS-C had fewer effector memory and more central memory cells than CSTs from convalescent adults (*p* < 0.0001). In **(A, C)** Boxes represent interquartile ranges; lines: median values; whiskers: minimum to maximum. In **(B)** Boxes represent medians; whiskers: 95% confidence interval. EM, effector memory; CM, central memory; TE, terminal effector. Asterisk (*) signifies *p* < 0.05.

To evaluate the phenotype and polyfunctionality of the CSTs, we measured their surface markers and performed cytokine Luminex assays. As shown in **Figure 5A**, there was a CD4+ T cell predominance in all cohorts with polyfunctional responses to spike, membrane and nucleocapsid antigens. The median percentage of CD4+ T cells in CSTs derived from adult, pediatric MIS-C, and pediatric non-MIS-C cohorts were 82.4% (95% CI = 75.4 – 88.5%), 71.95% (95% CI = 65.3 – 78.2%), and 72.7% (95% CI = 67.8 – 84.8%), respectively. CD8+ T cells comprised 5.9% (95% CI = 2.2 – 10.3%), 3.13% (95% CI = 1.67 – 7.22%), and 14.6% (95% CI = 3.78 – 22.5%) of CSTs derived from adult, pediatric MIS-C, and pediatric non-MIS-C cohorts, respectively. This CD4+ T cell predominance was consistent across viral antigens (**Figure 5B**). While both convalescent adults and children with MIS-C demonstrated a robust CST response, the memory phenotype of these CSTs differed, with a significantly higher percentage of central memory T cells and fewer effector memory T cells in MIS-C patients compared to convalescent adults (median 34.4% v 10.4% central memory and 51.6% v 84.2% effector memory, *p* < 0.0001) (**Figure 5C**). This memory phenotype differed between adults and children with and without MIS-C in CD4+ and CD8+ CST subsets (**Supplemental Figure 3**). Finally, supernatants from post-expansion lymphocytes from MIS-C patients (n = 17) re-stimulated with viral peptide pools for 48 hours exhibited higher concentrations of GM-CSF, IL-2, IL-4, IL-10, IL-13, TNF-α, and IL-2 receptor alpha over the actin baseline (*p* ≤ 0.03) (**Supplemental Figure 4**). Most of these cytokines were higher in supernatants from MIS-C samples compared to non-MIS-C (n = 3) but this difference did not meet statistical significance, possibly due to the small sample size (**Supplemental Figure 5**).

## Discussion

In this study, we sought to identify differences in SARS-CoV-2-specific T-cell responses in children with and without MIS-C versus adults with uncomplicated infection. We discovered that the pediatric CST response to SARS-CoV-2 in the absence of MIS-C is significantly weaker compared to the CST response observed in adult convalescent subjects and pediatric patients with MIS-C, a finding that points to differences in the virus-specific adaptive immune response among pediatric patients with MIS-C versus those without. However, convalescent adults displayed a stronger CST response than either pediatric group.

The stratification by age of T cell response to viral antigen is important in the setting of known age-related differences in illness severity with exposure to SARS-CoV-2 and suggests that SARS-CoV-2 may induce a more robust CST response in adults as compared to children. This finding adds to that published by Pierce et al. (30) in which T cells from adults compared to children post SARS-CoV-2 infection had more IFN-γ secretion and CD25 expression in response to the spike antigen, and establishes that not only spike but also membrane and nucleocapsid antigens induce a stronger CST response in adults compared to children post infection. It also suggests that children post infection, even despite seroconversion, have a proportionally lower T cell response to SARS-CoV-2 which may be due to the predominantly milder clinical course of SARS-CoV-2 infection in children compared with adults (1, 31). While both adult and pediatric cohorts reported mild to moderate disease, the pediatric cohort was generally milder with illness lasting 1-2 days consistent with reported outcomes for children versus adults with SARS-CoV-2 infection. Whether a more effective innate immune response to viral infection in children with uncomplicated SARS-CoV-2 infection explains these differences in adaptive immunity remains to be determined (30, 32–34). Additionally, the lower magnitude of T-cell responses in pediatric patients reflects the substantial need for COVID-19 vaccinations in all children.

In comparison to children with uncomplicated SARS-CoV-2 infection, the MIS-C cohort mounted a significantly more robust CST response to SARS-CoV-2 antigens. Importantly, while median time from acute infection could not be reliably calculated given that most MIS-C patients had no clear COVID-19 history (positive RNA test or symptoms), those who did have a known exposure, symptoms, or a positive RNA test reported that these events occurred 1-2 months prior to clinical presentation and sample collection. This is consistent with the timing of SARS-CoV-2 exposure relative to development of MIS-C in previous studies (3, 4, 8, 35) and suggests that the timing of our study evaluations of MIS-C patients may be more proximal to acute SARS-CoV-2 infection compared to our non-MIS-C pediatric and adult convalescent samples. It will therefore be informative to measure the CST response in children serially following MIS-C to determine the half-life of T cell responses. We also demonstrated that post recovery, patients with MIS-C elicited a robust adaptive immune response to SARS-CoV-2 despite receiving steroids and IL-1 blockade, suggesting that protective cell mediated immunity was preserved.

Our results suggest that children with MIS-C have a stronger T cell response to SARS-CoV-2 antigen than SARS-CoV-2 exposed children without MIS-C, despite no clear history of more severe primary SARS-CoV-2 illness than their non-MIS-C convalescent pediatric counterparts. The interplay between this response and immunopathology in MIS-C remains to be established. It has been suggested that chronic antigen exposure or delayed innate immune responses could contribute to the development of MIS-C (32, 36), which might lead to the higher CST frequency that we identified here. Multiple groups have also identified potentially pathologic immune cell subpopulations including non-virus-specific T cell clonotypes (Vbeta 21.3+) and activated CX3CR1+ T cells, along with putative autoantibodies to gastrointestinal and vascular proteins (7, 9, 11), which may indicate a post-infectious autoimmune etiology for the syndrome. Characterization of the interaction between CSTs and other activated T cell subsets may prove useful in identifying the underlying causes of MIS-C (11).

Similar to the results presented here, Hsieh et al. (10) also identified an intact CST response in patients with MIS-C with a similar pattern of antigen responsiveness compared to uncomplicated convalescent adults and children, though their study included a small number of children (11 with MIS-C and 2 without MIS-C). Pierce et al. (30) identified a weaker CST response in children compared to adults but did not compare the magnitude of response in children with and without MIS-C, likely also due to sample size (5 MIS-C patients).

This study has some limitations. First, because children acutely infected with SARS-CoV-2 often do not present to the hospital, the number of convalescent pediatric subjects in this analysis is low and their demographics differ from both the MIS-C and adult cohorts. Second, the heterogeneity in sampling times makes it difficult to directly compare the CST response in children with and without MIS-C. The small sample size precluded regression analysis to account for this. It will be essential to collect samples from MIS-C patients at later time points to determine whether the heightened CST response observed here is sustained over time. Relatedly, we cannot determine based on these data whether the higher CST response in MIS-C was due to the acute inflammatory milieu in MIS-C. Additionally, pediatric samples were drawn more proximally to acute infection than adult samples. Though we do not expect that this would cause the differences that we observed here, this should be confirmed with longitudinal analysis of pediatric samples. Lastly, we used a well-described strategy for the expansion and analysis of virus-specific T cells in this work. While this approach provides a sample enriched in virus-specific T cells, it also exposes samples to exogenous cytokines and peptides. Therefore, while we can reliably establish significant differences in CST frequency between the cohorts, we acknowledge that we cannot draw definite conclusions regarding potential phenotypic differences of *in vivo* bulk T cell phenotype in our subjects prior to CST expansion.

In summary, we have demonstrated significant differences in CST responses in pediatric subjects post uncomplicated SARS-CoV-2 infection versus convalescent adults and patients with MIS-C. Future longitudinal studies tracking immune responses over time will be essential to further interrogate the role of T-cells in the pathogenesis of inflammatory complications of COVID-19 in adults as well as children with MIS-C.

## Data Availability Statement

The original contributions presented in the study are included in the article/**Supplementary Material**. Further inquiries can be directed to the corresponding author.

## Ethics Statement

The studies involving human participants were reviewed and approved by Children’s National Hospital and National Institutes of Health Institutional Review Boards. Written informed consent to participate in this study was provided by the participant or the participant’s legal guardian.

## Author Contributions

Conceptualization: SC, CB, MK, JC, PB, MD, and RD. Methodology: SC, CB, MK, CL, JC, PB, and AZ. Investigation: SC, CL, NF, MJ-W, HL, VK, JD-S, HK, WS, KW, JC, PB, TI, ST, and AZ. Funding acquisition: MK, CB, JC, and PB. Project administration: HL, WS, KW, KS, ST, TI, MD, and RD. Writing – original draft: SC, CB, and MK. Writing – review & editing: SC, CB, MK, CL, NF, MJ-W, HL, VK, JD-S, HK, KW, JC, PB, ST, MD, RD, and AZ. All authors contributed to the article and approved the submitted version.

## Funding

This study was funded by the Board of Visitors of Children’s National Hospital, the Connor Family Foundation, the Katzen Foundation, the National Institute of Allergy and Infectious Diseases, and the National Institute of Dental and Craniofacial Research. Study sponsors had no role in study design, data collection, analysis, interpretation, or manuscript preparation.

## Conflict of Interest

CB is a co-founder and on the scientific advisory boards for Catamaran Bio and Mana Therapeutics with stock and/or ownership, is on the Board of Directors for Caballeta Bio with stock options and has stock in Neximmune and Repertoire Immune Medicines. MK is on a scientific advisory panel for Enzyvant.

The remaining authors declare that the research was conducted in the absence of any commercial or financial relationships that could be construed as a potential conflict of interest.

## Publisher’s Note

All claims expressed in this article are solely those of the authors and do not necessarily represent those of their affiliated organizations, or those of the publisher, the editors and the reviewers. Any product that may be evaluated in this article, or claim that may be made by its manufacturer, is not guaranteed or endorsed by the publisher.

## Abbreviations

MIS-C, multisystem inflammatory syndrome in children; CST, coronavirus-specific T cell; PBMCs, Peripheral blood mononuclear cells; COVID-19, 2019 coronavirus disease; LIPS, Luciferase immunoprecipitation systems.

## References

[1] Mehta NS Mytton OT Mullins EWS Fowler TA Falconer CL Murphy OB. SARS-CoV-2 (COVID-19): What Do We Know About Children? A Systematic Review. Clin Infect Dis (2020) 71(9):2469–79. doi: 10.1093/cid/ciaa556 PMC7239259 32392337

[2] Bi Q Wu Y Mei S Ye C Zou X Zhang Z. Epidemiology and Transmission of COVID-19 in 391 Cases and 1286 of Their Close Contacts in Shenzhen, China: A Retrospective Cohort Study. Lancet Infect Dis (2020) 20(8):911–9. doi: 10.1016/S1473-3099(20)30287-5 PMC7185944 32353347

[3] Feldstein LR Rose EB Horwitz SM Collins JP Newhams MM Son MBF. Multisystem Inflammatory Syndrome in U.S. Children and Adolescents. N Engl J Med (2020) 383(4):334–46. doi: 10.1056/NEJMoa2021680 PMC7346765 32598831

[4] Feldstein LR Tenforde MW Friedman KG Newhams M Rose EB Dapul H. Characteristics and Outcomes of US Children and Adolescents With Multisystem Inflammatory Syndrome in Children (MIS-C) Compared With Severe Acute COVID-19. JAMA (2021) 325(11):1074–87. doi: 10.1001/jama.2021.2091 PMC7905703 33625505

[5] Hoste L Van Paemel R Haerynck F. Multisystem Inflammatory Syndrome in Children Related to COVID-19: A Systematic Review. Eur J Pediatr (2021) 180(7):2019–34. doi: 10.1007/s00431-021-03993-5 PMC7890544 33599835

[6] DeBiasi RL Harahsheh AS Srinivasalu H Krishnan A Sharron MP Parikh K. Multisystem Inflammatory Syndrome of Children: Sub-Phenotypes, Risk Factors, Biomarkers, Cytokine Profiles and Viral Sequencing. J Pediatr (2021) 237:125–35.e18. doi: 10.1056/NEJMoa2021680 34181987

[7] Consiglio CR Cotugno N Sardh F Pou C Amodio D Rodriguez L. The Immunology of Multisystem Inflammatory Syndrome in Children With COVID-19. Cell (2020) 183(4):968–981 e7. doi: 10.1016/j.cell.2020.09.016 32966765 PMC7474869

[8] Verdoni L Mazza A Gervasoni A Martelli L Ruggeri M Ciuffreda M. An Outbreak of Severe Kawasaki-Like Disease at the Italian Epicentre of the SARS-CoV-2 Epidemic: An Observational Cohort Study. Lancet (2020) 395(10239):1771–8. doi: 10.1016/S0140-6736(20)31103-X PMC7220177 32410760

[9] Gruber CN Patel RS Trachtman R Lepow L Amanat F Krammer F. Mapping Systemic Inflammation and Antibody Responses in Multisystem Inflammatory Syndrome in Children (MIS-C). Cell (2020) 183(4):982–95.e14. doi: 10.1016/j.cell.2020.09.034 32991843 PMC7489877

[10] Hsieh LE Grifoni A Sidney J Shimizu C Shike H Ramchandar N. Characterization of SARS-CoV-2 and Common Cold Coronavirus-Specific T-Cell Responses in MIS-C and Kawasaki Disease Children. Eur J Immunol (2021) 2:10.1002/eji.202149556. doi: 10.1002/eji.202149556 PMC8646471 34599760

[11] Moreews M Le Gouge K Khaldi-Plassart S Pescarmona R Mathieu AL Malcus C. Polyclonal Expansion of TCR Vbeta 21.3(+) CD4(+) and CD8(+) T Cells is a Hallmark of Multisystem Inflammatory Syndrome in Children. Sci Immunol (2021) 6(59) 6(59):eabh1516. doi: 10.1126/sciimmunol.abh1516 34035116 PMC8815705

[12] Vella LA Giles JR Baxter AE Oldridge DA Diorio C Kuri-Cervantes L. Deep Immune Profiling of MIS-C Demonstrates Marked But Transient Immune Activation Compared to Adult and Pediatric COVID-19. Sci Immunol (2021) 6(57):eabf7570. doi: 10.1101/2020.09.25.20201863 33653907 PMC8128303

[13] Huang JJ Gaines SB Amezcua ML Lubell TR Dayan PS Dale M. Upregulation of Conventional Dendritic Cells Type 1 (Cdc1) Implicates Antigen Cross Presentation in Multisystem Inflammatory Syndrome (MIS-C). J Allergy Clin Immunol (2021) 22(21):01627–4. doi: 10.1016/j.jaci.2021.10.015 PMC8530782 34688775

[14] Syrimi E Fennell E Richter A Vrljicak P Stark R Ott S. The Immune Landscape of SARS-CoV-2-Associated Multisystem Inflammatory Syndrome in Children (MIS-C) From Acute Disease to Recovery. iScience (2021) 24(11):103215. doi: 10.1016/j.isci.2021.103215 34632327 PMC8487319

[15] Ahmad F Ahmed A Rajendraprasad SS Loranger A Gupta S Velagapudi M. Multisystem Inflammatory Syndrome in Adults: A Rare Sequela of SARS-CoV-2 Infection. Int J Infect Dis (2021) 108:209–11. doi: 10.1016/j.ijid.2021.05.050 PMC8142712 34044140

[16] Patel P DeCuir J Abrams J Campbell AP Godfred-Cato S Belay ED. Clinical Characteristics of Multisystem Inflammatory Syndrome in Adults: A Systematic Review. JAMA Netw Open (2021) 4(9):e2126456. doi: 10.1001/jamanetworkopen.2021.26456 34550381 PMC8459192

[17] Vardhana SA Wolchok JD. The Many Faces of the Anti-COVID Immune Response. J Exp Med (2020) 217(6):e20200678. doi: 10.1084/jem.20200678 32353870 PMC7191310

[18] Peng Y Mentzer AJ Liu G Yao X Yin Z Dong D. Broad and Strong Memory CD4(+) and CD8(+) T Cells Induced by SARS-CoV-2 in UK Convalescent Individuals Following COVID-19. Nat Immunol (2020) 21:1336–45. doi: 10.1101/2020.06.05.134551 PMC7611020 32887977

[19] Grifoni A Weiskopf D Ramirez SI Mateus J Dan JM Moderbacher CR. Targets of T Cell Responses to SARS-CoV-2 Coronavirus in Humans With COVID-19 Disease and Unexposed Individuals. Cell (2020) 181(7):1489–1501 e15. doi: 10.1016/j.cell.2020.05.015 32473127 PMC7237901

[20] Dan JM Mateus J Kato Y Hastie KM Yu ED Faliti CE. Immunological Memory to SARS-CoV-2 Assessed for Up to 8 Months After Infection. Science (2021) 371(6529):eabf4063. doi: 10.1126/science.abf4063 33408181 PMC7919858

[21] Braun J Loyal L Frentsch M Wendisch D Georg P Kurth F. SARS-CoV-2-Reactive T Cells in Healthy Donors and Patients With COVID-19. Nature (2020) 371(6529):eabf4063. doi: 10.1038/s41586-020-2598-9 32726801

[22] Rydyznski Moderbacher C Ramirez SI Dan JM Grifoni A Hastie KM Weiskopf D. Antigen-Specific Adaptive Immunity to SARS-CoV-2 in Acute COVID-19 and Associations With Age and Disease Severity. Cell (2020) 183(4):996–1012.e19. doi: 10.1016/j.cell.2020.09.038 33010815 PMC7494270

[23] Keller MD Harris KM Jensen-Wachspress MA Kankate V Lang H Lazarski CA. SARS-CoV-2 Specific T-Cells Are Rapidly Expanded for Therapeutic Use and Target Conserved Regions of Membrane Protein. Blood (2020) 136(25):2905–17. doi: 10.1182/blood.2020008488 PMC7746091 33331927

[24] Lucas C Wong P Klein J Castro TBR Silva J Sundaram M. Longitudinal Analyses Reveal Immunological Misfiring in Severe COVID-19. Nature (2020) 584(7821):463–9. doi: 10.1038/s41586-020-2588-y PMC7477538 32717743

[25] Mathew D Giles JR Baxter AE Oldridge DA Greenplate AR Wu JE. Deep Immune Profiling of COVID-19 Patients Reveals Distinct Immunotypes With Therapeutic Implications. Science (2020) 369(6508):eabc8511. doi: 10.1126/science.abc8511 32669297 PMC7402624

[26] Multisystem Inflammatory Syndrome in Children (MIS-C) Associated With Coronavirus Disease 2019 (COVID-19). Centers for Disease Control and Prevention Health Alert Network (2020). Available at: https://emergency.cdc.gov/han/2020/han00432.asp.

[27] Gerdemann U Keirnan JM Katari UL Yanagisawa R Christin AS Huye LE. Rapidly Generated Multivirus-Specific Cytotoxic T Lymphocytes for the Prophylaxis and Treatment of Viral Infections. Mol Ther (2012) 20(8):1622–32. doi: 10.1038/mt.2012.130 PMC3412490 22801446

[28] Burbelo PD Riedo FX Morishima C Rawlings S Smith D Das S. Detection of Nucleocapsid Antibody to SARS-CoV-2 is More Sensitive Than Antibody to Spike Protein in COVID-19 Patients. medRxiv (2020) 222(2):206–13. doi: 10.1101/2020.04.20.20071423 PMC7313936 32427334

[29] Thieme CJ Anft M Paniskaki K Blazquez-Navarro A Doevelaar A Seibert FS. Robust T Cell Response Toward Spike, Membrane, and Nucleocapsid SARS-CoV-2 Proteins Is Not Associated With Recovery in Critical COVID-19 Patients. Cell Rep Med (2020) 1(6):100092. doi: 10.1016/j.xcrm.2020.100092 32904468 PMC7456276

[30] Pierce CA Preston-Hurlburt P Dai Y Aschner CB Cheshenko N Galen B. Immune Responses to SARS-CoV-2 Infection in Hospitalized Pediatric and Adult Patients. Sci Transl Med (2020) 12(564):eabd5487. doi: 10.1126/scitranslmed.abd5487 32958614 PMC7658796

[31] Long QX Jia YJ Wang X Deng HJ Cao XX Yuan J. Immune Memory in Convalescent Patients With Asymptomatic or Mild COVID-19. Cell Discov (2021) 7(1):18. doi: 10.1038/s41421-021-00250-9 33767156 PMC7993859

[32] Rowley AH. Understanding SARS-CoV-2-Related Multisystem Inflammatory Syndrome in Children. Nat Rev Immunol (2020) 20(8):453–4. doi: 10.1038/s41577-020-0367-5 PMC7296515 32546853

[33] Bastard P Rosen LB Zhang Q Michailidis E Hoffmann HH Zhang Y. Autoantibodies Against Type I IFNs in Patients With Life-Threatening COVID-19. Science (2020) 370(6515):eabd4585. doi: 10.1126/science.abd4585 32972996 PMC7857397

[34] Hadjadj J Yatim N Barnabei L Corneau A Boussier J Smith N. Impaired Type I Interferon Activity and Inflammatory Responses in Severe COVID-19 Patients. Science (2020) 369(6504):718–24. doi: 10.1126/science.abc6027 PMC7402632 32661059

[35] Capone CA Subramony A Sweberg T Schneider J Shah S Rubin L. Characteristics, Cardiac Involvement, and Outcomes of Multisystem Inflammatory Syndrome of Childhood Associated With Severe Acute Respiratory Syndrome Coronavirus 2 Infection. J Pediatr (2020) 224:141–5. doi: 10.1016/j.jpeds.2020.06.044 PMC7293762 32553873

[36] Martinez OM Bridges ND Goldmuntz E Pascual V. The Immune Roadmap for Understanding Multi-System Inflammatory Syndrome in Children: Opportunities and Challenges. Nat Med (2020) 26(12):1819–24. doi: 10.1038/s41591-020-1140-9 33139949

