# Supplementary material for: SARS-CoV-2-Specific T Cell Responses Are Stronger in Children With Multisystem Inflammatory Syndrome Compared to Children With Uncomplicated SARS-CoV-2 Infection
Source: Front Immunol. 2022 Jan 18;12:793197. doi: 10.3389/fimmu.2021.793197 (PMC8803660; doi:10.3389/fimmu.2021.793197)
Supplement: Supplementary file 1 [file DataSheet_1.pdf]

Supplementary Materials

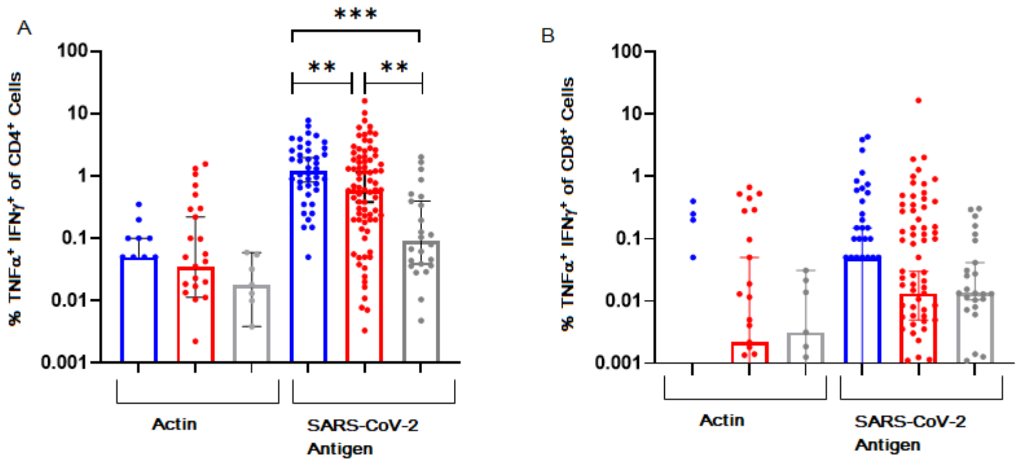

Supplemental Figure 1. CST response among CD4+ and CD8+ T cell subsets. Percent of (**A**) CD4+ and (**B**) CD8+ T lymphocytes from convalescent adult (blue), non-MIS-C pediatric (gray), and MIS-C (red) subjects secreting TNF-α and IFN-γ when stimulated with actin (negative control) or viral antigen post expansion in the presence of viral peptides. Actin = negative control. ***: $p < 0.0001$, **: $p < 0.05$. Column = median, error bars = 95% CI.

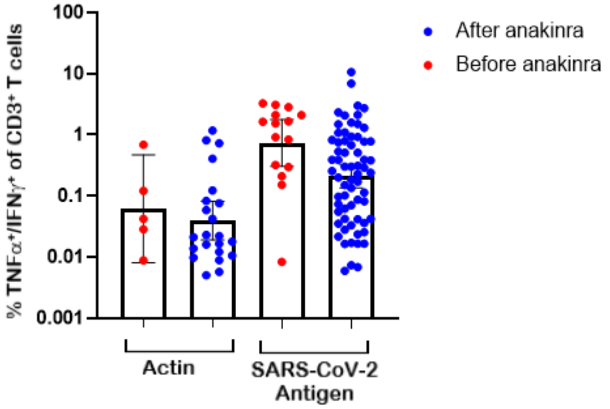

Supplemental Figure 2. Effect of anakinra on CST expansion. Magnitude of CST expansion from samples drawn before (red) and after (blue) anakinra treatment (1.39% versus 0.78%, p = 0.16). Boxes: geometric mean, whiskers: 95% CI around geometric mean.

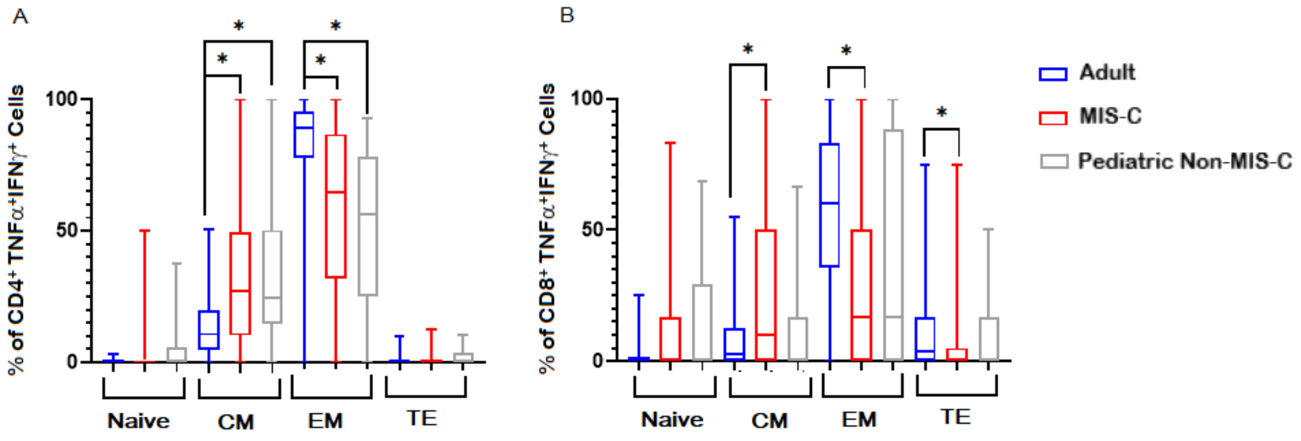

Supplemental Figure 3. Memory phenotype of CST Subsets. Percent of **(A)** CD4+ and **(B)** CD8+ CSTs expressing markers of T cell memory consistent with naïve ($CCR7^{+}CD45RO^{-}$), central memory (CM) ($CCR7^{+}CD45RO^{+}$), effector memory (EM) ($CCR7^{-}CD45RO^{+}$), and terminal effector (TE) ($CCR7^{-}CD45RO^{-}$) phenotype. Boxes: interquartile range, lines: median, whiskers: minimum to maximum, *: $p < 0.05$.

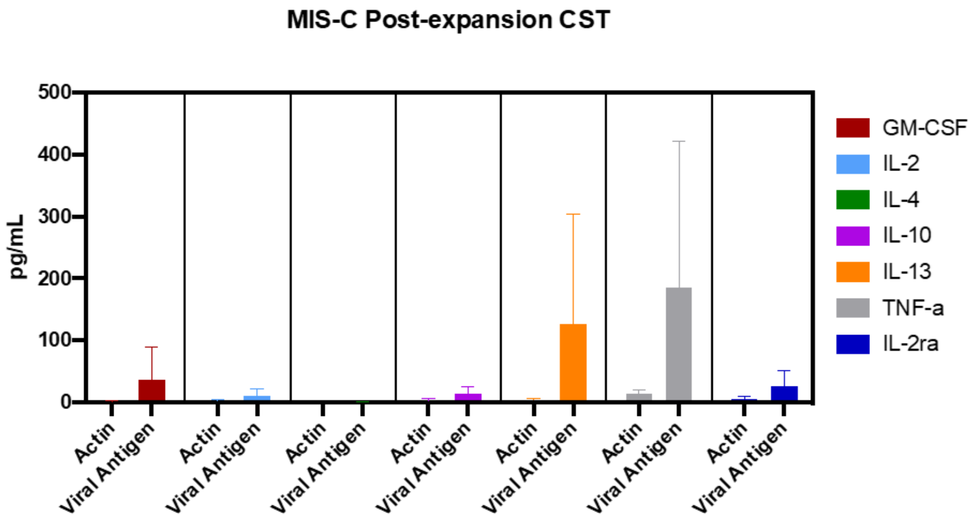

Supplemental Figure 4. MIS-C sample post-expansion cytokine secretion. SARS-CoV-2-specific cytokine production in CSTs from MIS-C patients following 10-day expansion. Differences between combined SARS-CoV-2 antigen and actin restimulation were significant for all displayed cytokines ($p<0.05$). Boxes: mean values; whiskers: standard deviation.

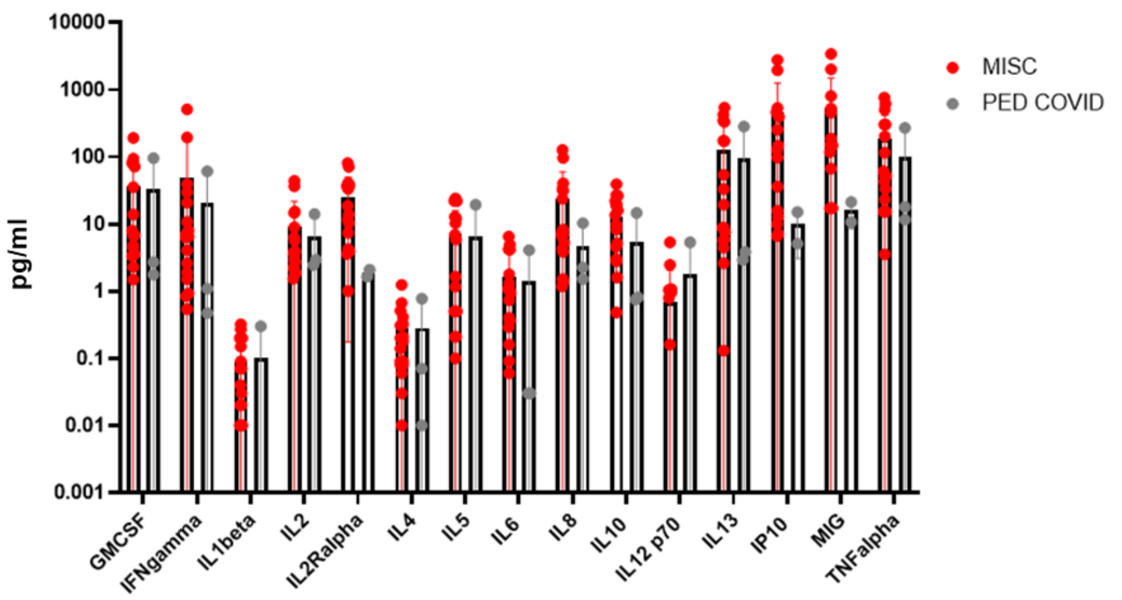

Supplemental Figure 5. MIS-C versus non-MIS-C pediatric post-expansion cytokine secretion. Comparison of SARS-CoV-2-specific cytokine production in pediatric patients with MIS-C (red) vs uncomplicated COVID-19 (gray) following 10-day expansion. Differences were not statistically significant. Boxes: mean values; whiskers: standard deviation.

	HLA-A	HLA-A	HLA-B	HLA-B	HLA-C	HLA-C	DRB1	DRB1	DRB3	DRB3	DRB4	DRB4	DRB5	DQB1	DQB1	DQA1	DQA1	DPB1	DPB1	DPA1	DPA1
M	02:07:01:01	11:02:01:01	46:01:01:01	46:01:01:01	01:02:01:01	01:02:01:01	09:01:02:01	14:04:01:01	02:02:01:06		01:03:01:05			03:03:02:02	06:01:01:01	01:04:01:01	03:02:01:02	02:01:02:01	05:01:01:01	01:03:01:01	02:02:02:01
M	23:17:01:01	31:01:02:13	53:01:01:01	58:02:01:01	04:01:01:01	06:02:01:01	07:01:01:01	13:01:01:01			01:03:01:01			02:02:01:01	03:03:02:01	02:01:01:01	03:03:01:09	04:01:01:01	18:01:01:01	01:03:01:02	01:03:01:02
M	03:01:01:07	30:02:01:03	49:01:01:01	57:03:01:01	07:01:01:01	07:18:01:01	15:03:01:01	15:78					01:01:01:02	06:02:01:01	06:02:01:01	01:02:01:01	01:02:01:01	18:01:01:01	18:01:01:01	01:03:01:02	01:03:01:02
M	02:01:01:01	30:02:01:02	45:01:01:01	45:01:01:01	16:01:01:01	16:01:01:02	01:02:01:01	08:06						03:01:01:05	05:01:01:01	01:01:02:01	05:05:01:03	01:01:01:01	18:01:01:01	01:03:01:02	02:02:02:04
M	02:01:01:01	23:01:01:01	07:02:01:01	58:11	07:02:01:01	07:02:01:01	03:02:01:01	11:02:01:01						03:01:01:16	04:02:01:08	04:01:01:04	05:05:01:13	01:01:01:01	01:01:01:01	02:02:02:04	02:02:02:04
M	68:01:01:02	68:01:01:02	15:03:01:02	52:01:02:01	02:10:01:01	16:01:01:01	03:02:01:01	10:01:01:01						04:02:01:08	05:01:01:02	01:05:01:01	04:01:01:04	01:01:01:01	131:01:01:01	02:01:01:14	02:01:08:03
M	24:02:01:01	34:02:01:01	07:02:01:01	44:02:01:01	05:01:01:02	15:05:02:01	01:01:01:01	15:03:01:01					01:01:01:02	05:01:01:03	06:02:01:01	01:01:01:01	01:02:01:01	13:01:01:01	105:01:01:01	02:01:01:06	03:01:01:05
M	01:01:01:01	29:02:01:01	40:02:01:01	44:03:01:01	03:05	04:01:01:01	04:07:01:01	07:01:01:01			01:03:01:02 N	01:03:02		03:02:01:01	03:03:02:01	02:01:01:01	03:01:01:01	04:01:01:01	04:02:01:02	01:03:01:02	01:03:01:05
M	01:01:01:01	03:01:01:01	14:02:01:01	40:01:01:01	08:02:01:01	17:01:01:05	11:02:01:01	15:01:01:01	02:02:01:01					03:19:01:01	06:02:01:01	01:02:01:10	05:05:01:13	04:01:01:01	04:01:01:01	01:03:01:04	01:05
M	24:02:01:01	68:03:01:01	35:01:01:02	35:17:01	04:01:01:01	07:02:01:01	01:01:01:01	14:06:01						03:01:01:01	05:01:01:03	01:01:01:01	05:03:01:01	04:02:01:02	13:01:01:02	01:03:01:05	02:02:02:01
M	02:01:01:01	32:01:01:01	35:08:01:01	50:01:01:01	04:01:01:06	06:02:01:02	03:01:01:01	07:01:01:01			01:03:01:02 N			02:01:01:01	03:03:02:01			01:01:01:01	04:01:01:01	01:03:01:04	02:01:02:02
M	24:02:01:01	26:01:01:01	40:02:01:01	45:01:01:01	03:05	06:02:01:03	01:01:01:01	04:07:01:01			01:03:01:01			03:02:01:01	05:01:01:03	01:01:01:01	03:01:01:01	02:01:02:10	04:02:01:02	01:03:01:02	01:03:01:05
M	02:01:01:01	68:01:01:02	35:03:01:01	55:01:01:01	03:03:01:01	04:01:01:01	8:01:01	16:01:01						04:02:01:04	05:02:01:01	01:02:02:01	04:01:01:01	03:01:01:01	04:01:01:01	01:03:01:02	01:03:01:03
M	29:02:01:01	33:01:01:01	14:02:01:01	58:01:01:01	07:18:01:01	08:02:01:01	01:02:01:01	08:04:01						04:02:01:08	05:01:01:01	01:01:02:01	04:01:02:01	04:01:01:27	104:01:01:01	01:03:01:02	01:03:01:14
M	02:05:01:01	24:38	35:01:01:02	35:02:01:01	03:04:01:02	04:01:01:06	04:07:01:01	11:04:01:01	02:02:01:02		01:03:01:01			03:01:01:02	03:02:01:01	03:01:01:01	05:05:01:01	04:02:01:02	14:01:01:01	01:03:01:05	02:01:01:02
M	02:01:01:01	24:02:01:01	07:02:01:01	35:17:01	04:01:01:01	07:02:01:03	04:07:01:01	15:01:01:01			01:03:01:01			03:02:01:01	06:02:01:01	01:02:01:01	03:01:01:01	04:02:01:02	05:01:01:01	01:03:01:05	02:02:02:01
M	01:01:01:01	02:01:01:01	35:02:01:01	35:12:01:01	04:01:01:01	04:01:01:06	08:02:01:01	11:04:01:01	02:02:01:02	02:02:11				04:02:01:04	06:03:01:01	01:03:01:02	04:01:01:01	02:01:02:01	04:02:01:02	01:03:01:05	02:01:01:02
M	02:01:01:01	68:03:01:01	35:01:01:02	35:17:01	04:01:01:01	07:02:01:01	04:07:01:01	04:11:01			01:03:01:01			03:02:01:01	04:02:01:09	03:01:01:01	03:03:01:06	04:02:01:02	04:02:01:02	01:03:01:05	01:03:01:05
M	01:01:01:01	02:01:01:01	08:01:01:01	41:02:01:01	07:01:01:01	17:03:01:02												04:01:01:01	04:01:01:03	01:03:01:02	01:03:01:04
M	24:02:01:01	25:01:01:01	18:01:01:02	35:05:01:02	04:01:01:01	12:03:01:01	04:310	13:02:03			01:03:01:01		01:01:01:01	03:02:01:01	06:02:01:01	01:02:01:10	03:01:01:01	02:01:02:01	04:01:01:01	01:03:01:02	02:01:01:02
M	01:01:01:01	68:03:01:01	39:05:01:01	57:01:01:01	06:02:01:01	07:02:01:01	04:07:01:01	07:01:01:01			01:03:01:01	01:03:01:02 N		03:02:01:01	03:03:02:01	02:01:01:01	03:01:01:01	04:01:01:01	04:02:01:02	01:03:01:02	01:03:01:05
P	02:02:01:01	23:01:01:01	15:16:01:02	42:01:01:01	14:02:01:02	17:01:01:02	11:01:02:01	15:03:01:01	02:02:01:05				01:01:01:02	06:02:01:01	06:02:01:04	01:02:01:01	01:02:01:01	01:01:01:01	18:01:01:01	01:03:01:02	02:01:02:02
P	02:01:01:01	33:03:01:01	40:64:01:02	44:03:01:01	03:04:01:02	04:01:01:01	08:04:03	15:03:01:01					01:01:01:02	04:02:01:04	06:02:01:01	01:02:01:01	04:01:01:01	18:01:01:01	105:01:01:05	01:03:01:02	01:03:01:05
P	30:01:01:01	74:01:01:01	42:01:01:01	45:01:01:01	16:01:01:01	17:01:01:02	08:04:01	13:03:01:01						03:01:01:01	03:19:01:01	04:01:02:01	05:05:01:01	01:01:01:01	69:01:01:01	02:01:01:01	02:01:08:03
P	29:02:01:02	32:01:01:01	27:05:02:01	45:01:01:01	05:01:01:02	06:02:01:03	03:01:01:01	04:01:01:01			01:03:01:01			02:01:01:01	03:01:01:01	03:03:01:01	05:01:01:02	01:01:01:01	04:01:01:01	01:03:01:02	02:01:02:02
P	01:01:01:01	25:01:01:01	18:01:01:02	57:01:01:01	06:02:01:01	12:03:01:01	07:01:01:01	15:01:01:01			01:03:01:01	01:03:01:02 N	01:01:01:01	03:03:02:01	06:02:01:01	01:02:01:01	02:01:01:01	02:01:02:01	23:01:01:01	01:03:01:01	01:03:01:04
P	24:02:01:01	68:02:01:01	51:01:01:01	57:03:01:02	07:01:02:01	15:02:01:01	03:02:01:01	11:01:01:01	02:02:01:02	03:01:01:01				03:01:01:03	04:02:01:08	04:01:01:04	05:05:01:01	01:01:01:01	04:01:01:01	01:03:01:04	02:02:02:04
P	03:01:01:01	26:01:01:01	07:02:01:01	40:02:01:01	02:02:02:01	07:02:01:03	11:04:01:01	13:01:01:01	01:76	02:02:01:02				03:01:01:03	06:03:01:01	01:03:01:02	05:05:01:01	02:02:01:01	104:01:01:01	01:03:01:01	01:03:01:02

Supplemental Table 1. HLA Sequences. Traditional HLA allele sequences for MIS-C (N = 21) and non-MIS-C (N = 7) pediatric subjects. M = MIS-C, P = pediatric non-MIS-C.
